# TL1A/DR3 axis involvement in the inflammatory cytokine network during pulmonary sarcoidosis

**DOI:** 10.1186/s12948-015-0022-z

**Published:** 2015-08-03

**Authors:** M. Facco, A. Cabrelle, F. Calabrese, A. Teramo, F. Cinetto, S. Carraro, V. Martini, F. Calzetti, N. Tamassia, M. A. Cassatella, G. Semenzato, C. Agostini

**Affiliations:** Department of Medicine, Hematology and Clinical Immunology Branch, Padua University School of Medicine, Padua, Italy; Venetian Institute of Molecular Medicine, Padua, Italy; Department of Cardiac, Thoracic and Vascular Sciences, Padua University School of Medicine, Padua, Italy; Department of Pathology and Diagnostics, Section of General Pathology, School of Medicine, University of Verona, Verona, Italy

**Keywords:** TL1A/DR3, TNF family members, MMP-9, TIMP-1, Lung, Sarcoidosis

## Abstract

**Background:**

TNF-like ligand 1A (TL1A), a recently recognized member of the TNF superfamily, and its death domain receptor 3 (DR3), firstly identified for their relevant role in T lymphocyte homeostasis, are now well-known mediators of several immune-inflammatory diseases, ranging from rheumatoid arthritis to inflammatory bowel diseases to psoriasis, whereas no data are available on their involvement in sarcoidosis, a multisystemic granulomatous disease where a deregulated T helper (Th)1/Th17 response takes place.

**Methods:**

In this study, by flow cytometry, real-time PCR, confocal microscopy and immunohistochemistry analyses, TL1A and DR3 were investigated in the pulmonary cells and the peripheral blood of 43 patients affected by sarcoidosis in different phases of the disease (29 patients with active sarcoidosis, 14 with the inactive form) and in 8 control subjects.

**Results:**

Our results demonstrated a significant higher expression, both at protein and mRNA levels, of TL1A and DR3 in pulmonary T cells and alveolar macrophages of patients with active sarcoidosis as compared to patients with the inactive form of the disease and to controls. In patients with sarcoidosis TL1A was strongly more expressed in the lung than the blood, i.e., at the site of the involved organ. Additionally, zymography assays showed that TL1A is able to increase the production of matrix metalloproteinase 9 by sarcoid alveolar macrophages characterized, in patients with the active form of the disease, by reduced mRNA levels of the tissue inhibitor of metalloproteinase (TIMP)-1.

**Conclusions:**

These data suggest that TL1A/DR3 interactions are part of the extended and complex immune-inflammatory network that characterizes sarcoidosis during its active phase and may contribute to the pathogenesis and to the progression of the disease.

## Introduction

Sarcoidosis is a multisystemic granulomatous disease of unknown etiology characterized by a compartmentalization of CD4+ T helper 1 (Th1)/Th17 lymphocytes [1] and activated monocyte/macrophages in involved organs, predominantly the lungs [2, 3]. In the majority of patients the disease spontaneously resolves, but in some subjects, the persistence of the antigenic stimulus favors a chronic immuno-inflammatory state, the formation of lung granulomas, and, in some cases, an evolution toward fibrosis [4]. A redundant and complex network of cytokines and chemokines directs the pathogenesis of sarcoidosis: in the early phases, the sites of active disease are characterized by an overproduction of Th1/Th17 cytokines, such as interleukin (IL)-2, IFN-gamma [5], and IL-17 [1], associated with the high expression of macrophage-derived molecules like IL-15 [5], CXCL16 [6], CXCL10 [7], CCL20 [8], and CCL5 [9].

TNF-like ligand 1A (TL1A) and its death domain receptor 3 (DR3) are members of the tumor necrosis factor superfamily of ligands and receptors, respectively. Well-established and emerging evidence demonstrates the relevant impact of TL1A/DR3 signalling on the fate of a variety of immune cells, from T helper (1, 2, and 17) lymphocytes, to natural killer cells to monocytes [10], supporting the concept that TL1A is a crucial regulator of inflammation at the interface between the innate and adaptive immune system [11]. Particularly, the TL1A binding to DR3, mainly expressed on T cells, initiates a number of immune responses culminating in the activation of T cells and the secretion of pro-inflammatory mediators [12]. As a consequence of these effects, TL1A and its receptor are frequently involved in the pathogenesis of many autoimmune and inflammatory diseases, including rheumatoid arthritis [13, 14], psoriasis [15], and inflammatory bowel diseases [16, 17], most of them characterized by a Th1/Th17 microenvironment as sarcoidosis [17–19].

Herein, we investigate the hypothesis that TL1A and DR3 are implicate in the pathogenesis and/or evolution of sarcoidosis by stratifying forty-three patients in different phases of the disease and comparing them to control subjects.

## Methods

### Study population

Forty-three patients with sarcoidosis were analyzed. In all cases, the diagnosis was made from a biopsy obtained either from the lungs or from lymph nodes and showing non-caseating epithelioid granulomas with no evidence of inorganic material known to cause granulomatous diseases.

The patients underwent bronchoalveolar lavage (BAL) fluid analysis. In particular, twenty-nine sarcoid patients presenting with an episode of pulmonary involvement were evaluated at the onset of the disease. They were defined as having a high intensity alveolitis (i.e., the active form of the disease) on the basis of the following characteristics: lymphocytic alveolitis (> than 30x10^3^ lymphocytes/ml); lung CD4 to CD8 ratio >4.0. The assessment of disease activity included BAL, clinical features, chest radiograph, lung function tests, high-resolution computed tomography, and routine blood studies. BAL samples were also obtained from fourteen patients with previously diagnosed pulmonary sarcoidosis who repeated BAL fluid analysis during their follow-up period. These patients had normal lung function, normal BAL fluid cell numbers, and no clinical signs of acute disease. No patient received immunosuppressive therapy for 6 months prior to the BAL execution.

Eight subjects were selected as controls for the BAL studies, evaluated for cough complaints without lung disease. They had normal physical examination, chest X rays, lung function tests, and BAL cell numbers.

Peripheral blood from patients with sarcoidosis and from eight healthy subjects was also included in the study. Written informed consent was obtained from each patient and from controls.

### Preparation of cell suspensions

Following administration of local anaesthesia, BAL was performed as previously described [20]. Briefly, a total of 150–200 ml of saline solution was injected via fiber-optic bronchoscopy, in 25 ml aliquots, with immediate vacuum aspiration after each aliquot. The fluid was filtered through gauze, and its volume was measured. The amount of injected fluid recovered was 77.3 ± 7.5 %. Cells recovered from the BAL fluid were washed 3 times with PBS, resuspended in endotoxin tested RPMI 1640 (Sigma Chemical Co., St. Louis, MO) supplemented with 20 mM HEPES and L-glutamine, 100 U/ml penicillin, 100 μg/ml streptomycin, and 10 % fetal calf serum (FCS), (ICN Flow, Costa Mesa, CA) and then counted. Alveolar macrophages (AMs), lymphocytes, neutrophils and eosinophils were differentially counted in cytocentrifuged smears stained with Wright-Giemsa, for a total count of 300 cells, according to morphological criteria.

AMs and BAL T-cells were purified from BAL cell suspensions by rosetting with neuraminidase-treated sheep red blood cells followed by Ficoll-Hypaque gradient separations, as previously described [1]. AMs were further enriched by removing residual CD3+, CD16+, and CD56+ lymphocytes with magnetic separation columns (MiniMACS, Miltenyi Biotec), as previously described [1]. Staining with monoclonal antibodies (mAbs) showed that, after this multistep selection procedure, >98 % of AMs expressed the AM-associated CD68 antigen, whereas >98 % of the rosetting population was constituted by CD3+ T cells. CD4+ T cells were separated from CD8+ T lymphocytes by magnetic separations over columns (Mini MACS, Sunnyvale, CA), as previously reported [1].

Peripheral blood mononuclear cells (PBMCs) from the patients under study were obtained from freshly heparinized blood following centrifugation on Ficoll-Hypaque gradient and washing with PBS. Peripheral blood lymphocytes were further enriched following rosetting of PBMC with sheep-red-blood cells, as reported above, and CD4+ T-cells were separated from CD8+ T lymphocytes by magnetic separations over columns (Mini MACS).

### Monoclonal antibodies and cytokines

The commercially available conjugated or unconjugated mAbs used belonged to the Becton Dickinson and Pharmingen (San Diego, CA) series and included: CD3, CD4, CD8, CD11c, CD14, CD16, CD19, CD45R0, CD45RA, CD68, IL4 and IFN-γ, and isotype-matched controls. Anti- mAbs were purchased from R&D Systems Inc. (Minneapolis, MN). Purified mouse IgG1 anti-human TL1A (clone 12 F11) was purchased from Human Genome Sciences (GlaxoSmithKline, Verona, Italy) while mouse IgG1 anti-human DR3/TNFRSF25 was from R&D (R&D Systems, Milan, Italy). These mAbs were used for flow cytometry and immunohistochemistry analyses. A secondary antibody PE-conjugated rat anti-mouse IgG1 (Caltag Laboratories, Burlingame, CA, USA) was used for flow cytometry analysis. The frequency of positive cells for TL1A and DR3 was determined by FACS analysis. Cells were acquired on FACSCanto analyzer (Becton-Dickinson) and data processed by FACSDiva software program (Becton-Dickinson).

### Confocal microscopy

T cells and AMs from BAL of patients with active (*n* = 4) and inactive (*n* = 4) sarcoidosis and controls (*n* = 3) were plated in polylisine coated glass for 15 min at +4 °C, with anti-TL1A (1:150) and anti-DR3 (1:150) mAbs, and fixed in 4 % paraformaldehyde for 10 min. To reveal positivity for the molecules, a FITC-conjugated rat anti-mouse IgG1 (1:200) was used. Background staining with FITC-conjugated rat anti-mouse IgG1 alone was routinely compared with positively stained cells and was not visible using identical acquisition settings.

Slides were mounted with cover slips and fluorescence was detected using the UltraView LCI confocal system equipped with a fluorescence filter set for excitation at 488 nm.

### Western blot analysis

BAL cells (0.25 × 10^6^ for each sample) purified from 8 patients affected by active sarcoidosis, 7 patients with inactive disease, and 8 controls, were prepared by cell lyses with Tris 20 mM, NaCl 150 mM, EDTA 2 mM, EGTA 2 mM, Triton X-100 0.5 % supplemented with complete protease inhibitor cocktail (Roche; Mannheim, Germany) and sodium orthovanadate 1 mM (Calbiochem; Gibbstown, NJ). Samples were then subjected to SDS/PAGE (10 % gels), transferred to nitrocellulose membranes, and immunostained with goat polyclonal Ab anti-human TL1A/TNFS15 (R&D Systems Inc.), mouse mAb anti-human DR3/TNFRSF25 (R&D Systems Inc.), and mouse mAb anti-human β-actin (Sigma-Aldrich), using an enhanced chemiluminescent detection system (Pierce; Rockford, IL).

### Immunohistochemical analysis

Lung samples from eight cases of sarcoidosis (five from active, three from inactive forms, the last obtained from native lungs of patients requiring lung transplantation) were processed by immunohistochemistry.

Briefly four μm-thick sequential serial sections were pre-treated by boiling in citrate buffer (pH 6.1) in a microwave (700 W, 1 minute) for antigen retrieval. Afterwards, sections were treated with normal serum (Immunotech, Marseille, France) and incubated for 60 min with the primary monoclonal antibodies anti-IL 17 and anti-IL23R at a concentration of 1:20 and 1:50. Sections were subsequently incubated with rabbit horseradish peroxidase (HRP) polymer (Dako, Glostrup, Denmark) for 30 min. Immunoreactivity was visualized with 3-3’-diaminobenzidine (DAB, Dako, Glostrup, Denmark). Negative controls for non-specific binding were processed omitting the primary antibodies and revealed no signal.

### Real-Time PCR expression analysis

Total cellular RNA was extracted from cells using the RNeasy Mini Kit (Qiagen, Hilden, Germany) according to the manufacturer’s protocol and was treated with DNase (Qiagen). Complementary DNA was generated from 1 μg of total RNA using oligo-dT primer and the AMV reverse transcriptase (Promega, Madison, WI). Real-time PCR was carried out in an ABI Prism 7000 sequence detection system (Applied Biosystems, Foster City, CA). SYBR Green PCR Master Mix was purchased from Applied Biosystems. Real-Time PCR for TL1A, DR3, MMP-9 and TIMP-1 gene expression was performed in purified AMs and BAL T cells of patients with active (*n* = 25) and inactive sarcoidosis (*n* = 13). TL1A and DR3 mRNA levels were also evaluated in monocytes and T cells from the peripheral blood of the same patients and 8 healthy controls. The primers used were: TL1A: forward 5’-CAC CTC TTA GAG CAG ACG GAG ATA A-3’, reverse 5’-TTA AAG TGC TGT GTG GGA GTT TGT-3’; DR3: forward 5’-ACC CAT CTG TCA CCC TTG GA-3’, reverse 5’-CTG GAC GGT GCA GAT CTT CTC-3’; MMP-9: forward 5’-TGC CCG GAC CAA GGA TAC AG-3’, reverse 5'- GTG CAT TCC TCA CAG CCA ACA G; glyceraldehyde-3-phosphate dehydrogenase (GAPDH): forward 5’-AAT GGA AAT CCC ATC ACC ATC T-3’; reverse 5’-CGC CCC ACT TGA TTT TGG-3’. The primers were designed in our laboratory, whereas for TL1A we used the primers as described by Migone et al. [21]. Standard curves were generated for each gene. The relative amounts of messenger RNA (mRNA) were normalized for GAPDH expression.

### Gelatin zymography assays

This is an in vitro assay using gelatin-substrate gel electrophoresis we employed to measure the level of MMP-9 activity in BAL fluid components or in 24 h culture medium recovered from AMs and lung T cells, of patients with active (*n* = 7) and inactive (*n* = 6) sarcoidosis, cultured with or without TL1A (100 ng/ml, R&D Systems). BAL fluid components and conditioned supernatants were mixed with an equal volume of sample buffer (62.5 mM Tris–HCl, pH 6.8, 10 % glycerol, 2 % SDS, and 0.00625 % (w/v) bromophenol blue). Samples were electrophoresed on 7.5 % SDS polyacrylamide gel containing 2 mg/mL gelatin (type A, Sigma-Aldrich). After electrophoresis, the gel was washed three times for 30 min in 2.5 % Triton X-100 at room temperature, and incubated for 16 h at 37 °C in incubation buffer (50 mM Tris–HCl, pH 7.6, 5 mM CaCl2, 200 mM NaCl). The gel was stained for 30 min with Coomassie Brilliant Blue R-250 (Amersham Biosciences) and destained in washing solution (30 % methanol, 10 % acetic acid). White bands on the blue background represented gelatin digestion corresponding to the presence/activity of MMP-9. The bands were quantified by the image analysis software QuantityOne (Bio-Rad).

### Statistical analysis

Statistical analysis was performed using Student's *t* test, Kolgomorov-Smirnov analysis, and ANOVA. Data were expressed as mean ± standard deviation (SD) and were considered statistically significant when p values were <0.05.

## Results

### Patient characteristics

Twenty-nine subjects with high intensity alveolitis were evaluated at the onset of the disease (19 men and 10 women; mean age 42.5 ± 12.9 years; 7 smokers). Twenty-six patients required corticosteroid therapy; three patients spontaneously resolved. Fourteen patients (5 men and 9 women; mean age 40.5 ± 12.5 years; 2 smokers), with previously diagnosed pulmonary sarcoidosis, repeated BAL fluid analysis during their follow-up period (follow-up period average: 57.1 ± 18.6 months, range from 33–81 months). Further characteristics of patients studied are shown in Table 1. Eight subjects were selected as controls for the BAL studies (6 men and 2 women; mean age 37.5 ± 4.3 years; nonsmokers).

**Table 1:** Clinical characteristics of patients with sarcoidosis

Patients with active sarcoidosis (*n* = 29)	
Stage 1 (bilateral hilar lymphadenopathy)	13
Stage 2 (bilateral hilar lymphadenopathy with pulmonary infiltrates)	11
Stage 3 (parenchymal infiltrates without hilar adenopathy)	5
FVC%	103.33 ± 11.60
FEV1%	101.16 ± 14.76
DLCO%	79.33 ± 11.13
TIFF	92.00 ± 12.94
Patients with inactive sarcoidosis (*n* = 14)	
Stage 1	11
Stage 2	3
Stage 3	0
FVC%	98.20 ± 15.39
FEV1%	93.10 ± 17.99
DLCO%	86.77 ± 10.72
TIFF	80.01 ± 8.99

FVC, Forced vital capacity; FEV1, Forced Expiratory Volume in the first second; DLCO, Diffusing Capacity of the Lung for Carbon Monoxide; TIFF, Tiffeneau index

### Morphological and phenotypical analyses

Morphological and phenotypical features of cells obtained from the BAL of patients with sarcoidosis and eight control subjects are reported in Table 2. All subjects with active sarcoidosis showed a high intensity CD4+ lymphocytic alveolitis sustained by CD45RO+ T cells. The majority of these cells were equipped with the chemokine receptor CXCR3, IL-12Rβ, and intracytoplasmatic IFN-γ (data not shown). CD4+ and CD8+ T cell subsets and AMs detected in the BAL of patients with inactive sarcoidosis were superimposable to those observed in controls (Table 2).

**Table 2:** BAL characteristics of sarcoid patients and control subjects

	Cell recovery	Alveolar macrophages		Lymphocytes		CD4+ T cells		CD8+ T cells	
	x 10^3^/ml	x 10^3^/ml	%	x 10^3^/ml	%	x 10^3^/ml	%	x 10^3^/ml	%
active sarcoidosis (n: 29)	300 ± 37	208 ± 51	76 ± 10	79 ± 35	24 ± 9	69 ± 12	83 ± 7	13 ± 8	19 ± 6
inactive sarcoidosis (n: 14)	110 ± 23	109 ± 26	96 ± 4	6 ± 5	5 ± 2	4 ± 3	58 ± 2	3 ± 1	37 ± 4
controls (n: 8)	115 ± 17	111 ± 24	94 ± 3	5 ± 4	6 ± 3	4 ± 2	58 ± 6	3 ± 2	39 ± 7

### TL1A and DR3 expression in lung T cells and alveolar macrophages from patients with sarcoidosis

By flow cytometry, we investigated the presence of TL1A and DR3 on freshly obtained pulmonary and peripheral blood cells of patients with active and inactive sarcoidosis, and controls.

**Fig. 1:**
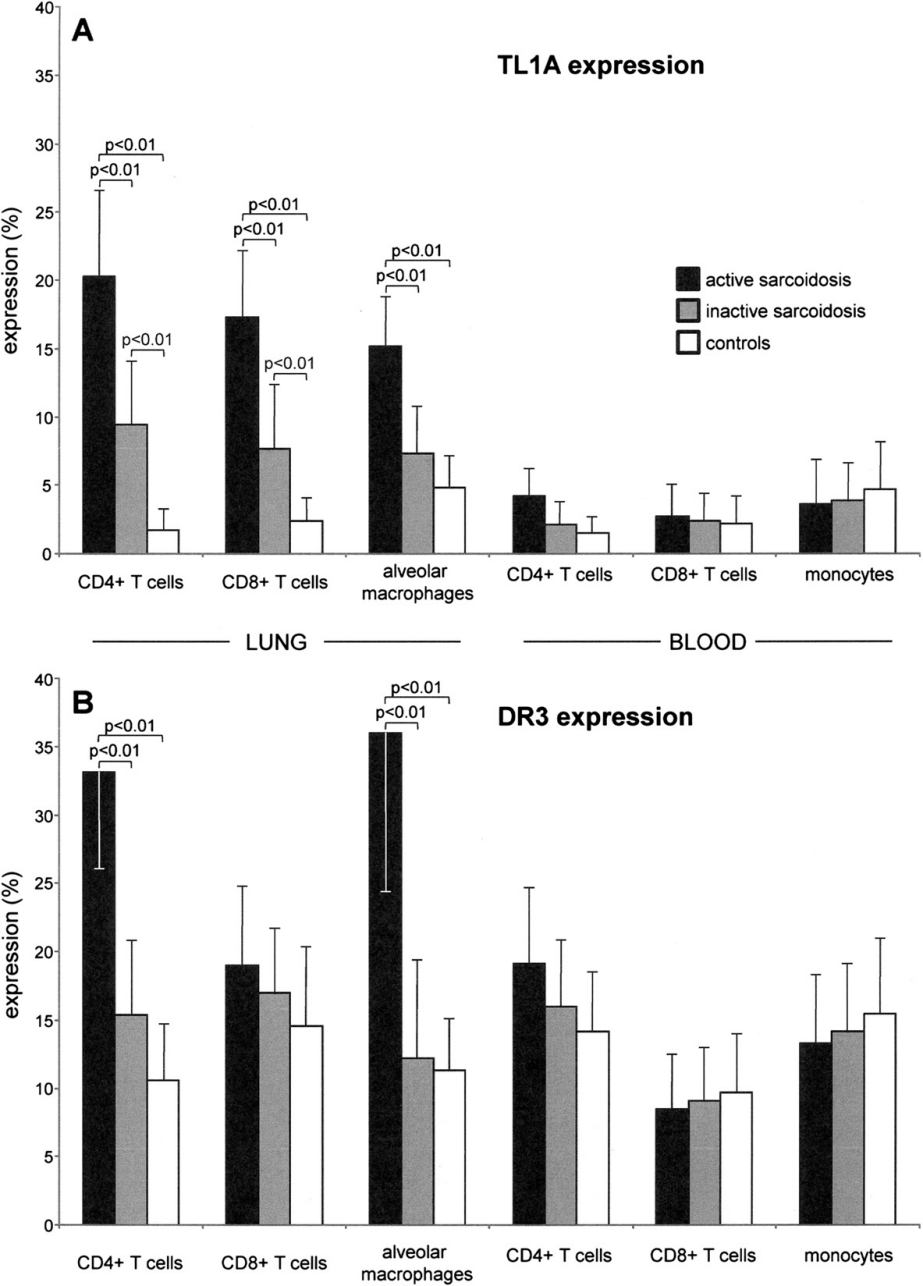
Flow cytometry analyses of TL1A (**a**) and DR3 (**b**) expression evaluated in T (CD4+ and CD8+) lymphocytes and alveolar macrophages/monocytes freshly obtained from the lung and the blood of patients affected by active sarcoidosis (black columns), inactive sarcoidosis (*grey columns*), and from control subjects (*white columns*). Data are expressed as mean ± SD

As shown in Fig. 1a, the percentage of freshly obtained pulmonary CD4+ T lymphocytes expressing TL1A was much higher in patients with the active form of the disease (20.3 % ± 6.3), as compared to inactive sarcoidosis (9.4 % ± 4.5 of CD4+ T cells; p < 0.01 vs active disease), and to controls (1.7 % ± 1.5 of CD4+ T lymphocytes; *p* < 0.01 vs active disease; ANOVA *p* < 0.01). CD8+ T cells showed a similar expression, characterized by more raised TL1A levels in patients with active sarcoidosis (17.3 % ± 4.9), as compared to inactive disease (7.7 % ± 4.7 of CD8+ T cells; *p* < 0.01 vs active disease), and to controls (2.4 % ± 1.6 of CD8+ T lymphocytes; p < 0.01 vs active disease; ANOVA *p* < 0.01). In addition, AMs were marked by a decreasing expression of TL1A from patients with the active form of disease (15.2 % ± 3.6), to inactive sarcoidosis (7.3 % ± 3.5 of AMs; *p* < 0.01 vs active disease), to controls (4.8 % ± 2.1 of AMs; *p* < 0.01 vs active disease; ANOVA *p* < 0.01) (Fig. 1a). Peripheral T cell populations and monocytes of patients and controls were characterized by low (with respect to lung) but, among them, comparable TL1A expressions, without any statistical difference (Fig. 1a). Mean fluorescence intensity (MFI) results, related to the amount of protein locates on cell surface, parallel the trends of TL1A expression in each different cell subset (data not shown).

As far as DR3 is concerned (Fig. 1b), lung CD4+ T cells obtained from patients with active sarcoidosis expressed the receptor in a percentage significantly higher (33.2 % ± 7.1) with respect to pulmonary CD4+ T cells of patients with inactive sarcoidosis (15.4 % ± 5.3; *p* < 0.01 vs active disease), and controls (10.6 % ± 4.1; *p* < 0.01 vs active disease; ANOVA *p* < 0.01; Fig. 1b). No relevant differences were found in DR3 expression by CD8+ T lymphocytes (active disease 19.0 % ± 5.8; inactive sarcoidosis 17.0 % ± 4.7; controls: 14.6 % ± 5.8, of CD8+ T cells; p: not significant), whereas AMs from patients with active sarcoidosis were marked out by the highest DR3 expression (36.0 % ± 11.7) as compared to inactive sarcoidosis (12.2 % ± 7.1 of AMs; *p* < 0.01 vs active disease), and to controls (11.3 % ± 3.8 of AMs; *p* < 0.01 vs active disease; ANOVA *p* < 0.01) (Fig. 1b).

In the peripheral blood, CD4 and CD8 T cell subsets and monocytes of patients and controls showed similar DR3 expression, with no significant differences (Fig. 1b).

As well as for TL1A, MFI data concerning DR3 followed the trend of the results described above (data not shown).

**Fig. 2:**
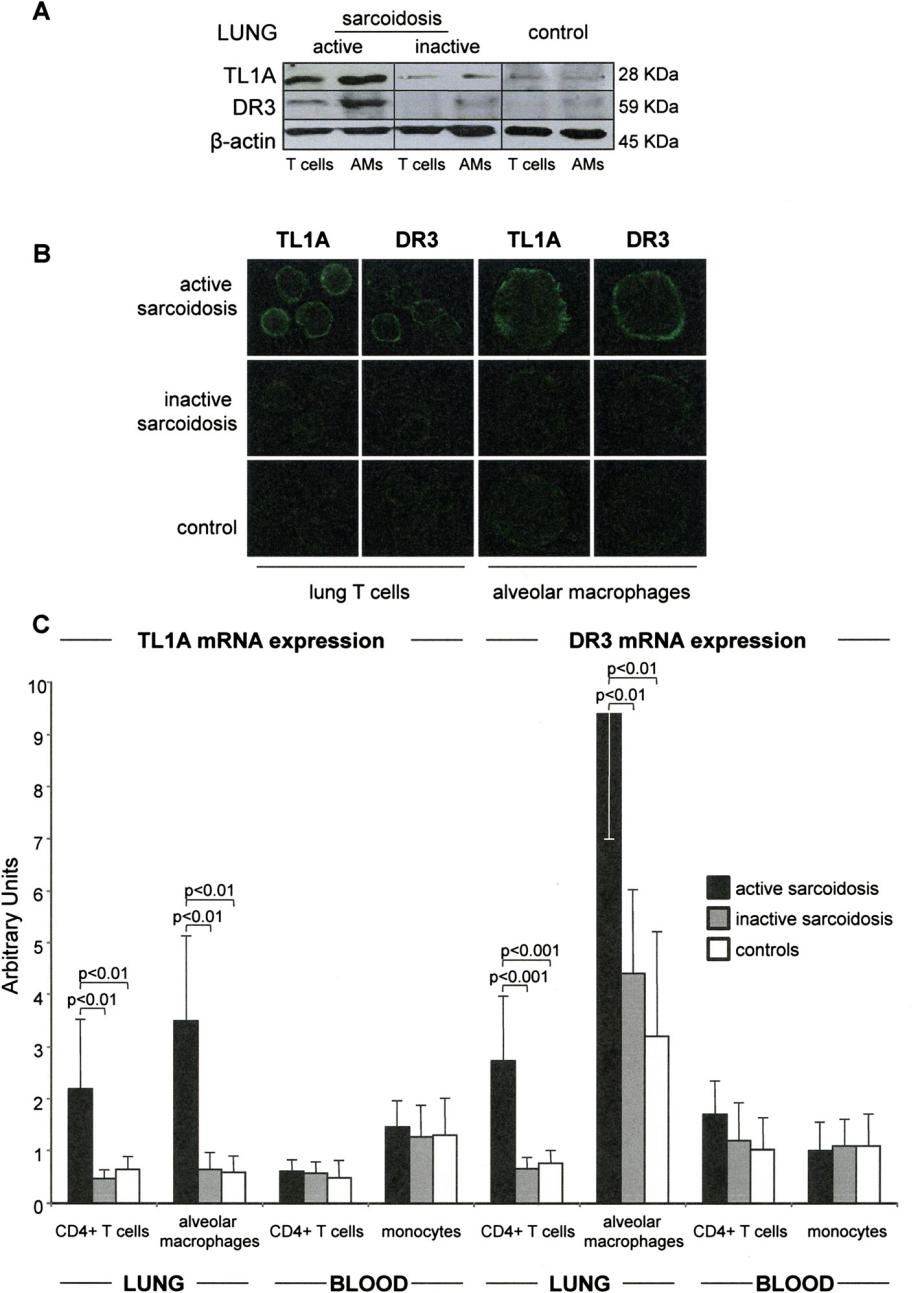
Western blotting (**a**) and confocal microscopy (**b**) analyses of TL1A and DR3 proteins evaluated in lung T cells and alveolar macrophages obtained from the BAL of two representative cases of patients with active and inactive sarcoidosis and of a control subject. (**c**) TL1A and DR3 mRNA expression in CD4+ T lymphocytes and alveolar macrophages/monocytes freshly obtained from the lung and the blood of patients affected by active sarcoidosis (*black columns*), inactive sarcoidosis (*grey columns*), and from control subjects (*white columns*). Expression of TL1A and DR3 mRNAs was normalized on GAPDH mRNA. Data are expressed as mean ± SD

Western blotting (Fig. 2a) and confocal microscopy analyses (Fig. 2b) of TL1A and DR3 confirmed the higher expression of the two molecules on lung T lymphocytes and alveolar macrophages obtained from patients with active sarcoidosis with respect to patients with the inactive form of the disease and to controls.

### Molecular analysis of TL1A and DR3 expression

As shown in Fig. 2c, real-time polymerase chain reaction evaluation of TL1A gene expression by highly purified BAL and blood CD4+ T lymphocytes, AMs and monocytes, demonstrated that mRNA expression of TL1A was higher in lung CD4+ T lymphocytes (2.2 ± 1.3) and AMs (3.5 ± 1.6) of active sarcoidosis than in the corresponding cell subsets derived from patients with inactive disease (CD4+ T cells: 0.46 ± 0.16; AMs: 0.64 ± 0.3; *p* < 0.01 vs active disease), and control subjects (CD4+ T cells: 0.64 ± 0.23; AMs: 0.59 ± 0.3; *p* < 0.01 vs active disease; ANOVA *p* < 0.01). Peripheral cell subsets of patients and controls were characterized by comparable TL1A mRNA expressions (p: not significant), (Fig. 2c).

Similarly, a real-time PCR evaluation of DR3 gene expression demonstrated that DR3 mRNA level was higher in lung CD4+ T lymphocytes (2.74 ± 1.2), and especially in AMs (9.40 ± 2.4) of active sarcoidosis than in the equivalent cell populations of patients with inactive disease (CD4+ T cells: 0.66 ± 1.2; *p* < 0.001 vs active disease; AMs: 4.4 ± 1.6; *p* < 0.01 vs active disease), and control subjects (CD4+ T cells: 0.78 ± 0.25; *p* < 0.001 vs active disease; AMs: 3.2 ± 2.0; *p* < 0.01 vs active disease). Again, no significant differences in DR3 mRNA expression stood out among peripheral cell subsets of patients and controls (p: not significant).

### TL1A and DR3 localization at sites of disease activity

**Fig. 3:**
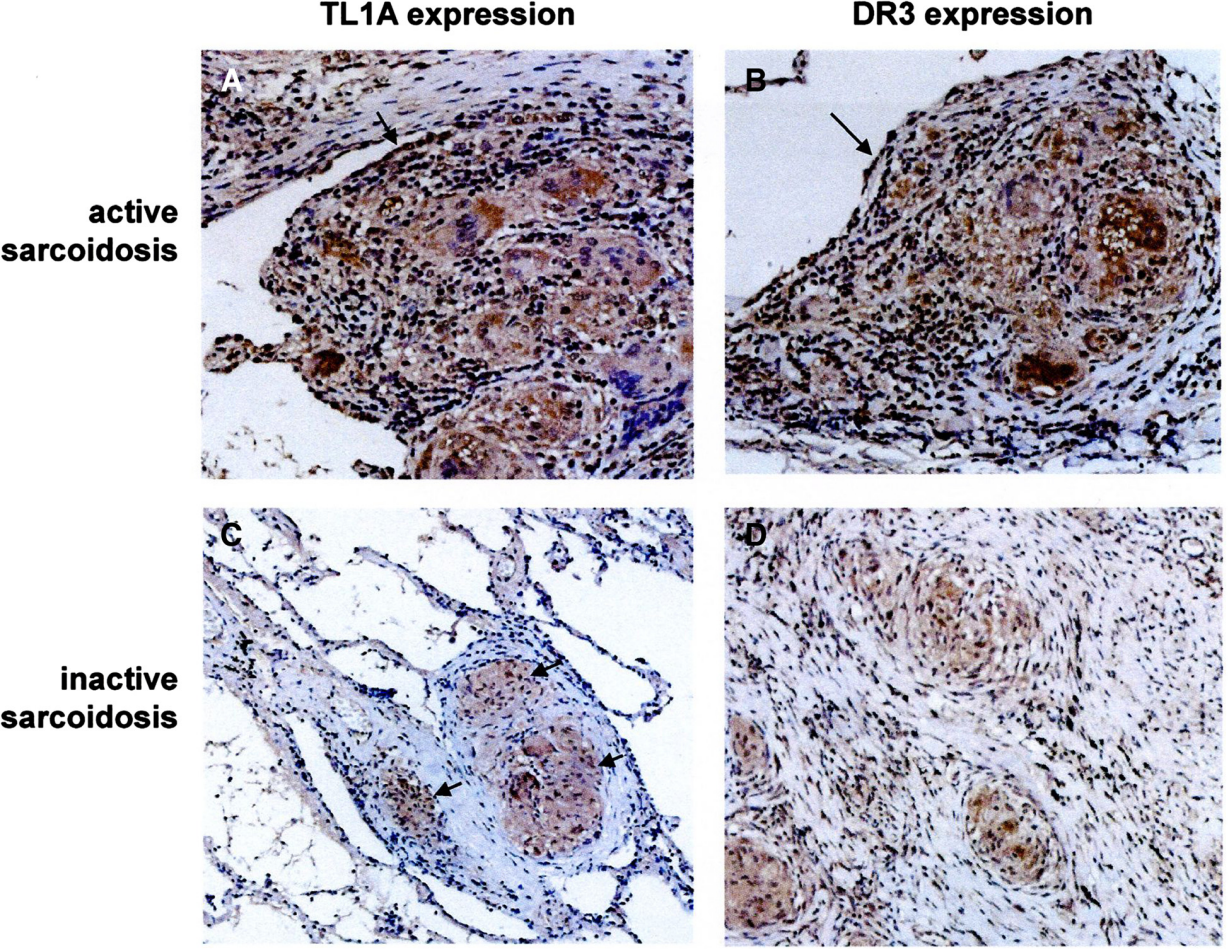
Immunohistochemistry for TL1A and DR3 expression in representative patients with active (A, B) and refractory sarcoidosis and pulmonary fibrosis (C, D). A, B. TL1A and DR3 are expressed at high intensity by macrophagic, both epitheliod and multinucleated, cells and lymphocytes infiltrating the lung biopsy of the patient with active sarcoidosis. The black arrows indicate positive metaplastic epithelial cells. C, D. In the lung specimen obtained from a patient with refractory sarcoidosis and pulmonary fibrosis, pulmonary cells were mainly non reactive for TL1A and DR3; a weak immunostaining was only seen in some epitheliod cells (*arrows*). Note marked fibrosis around the granulomatous nodule. Original magnification 400x

Immunohistochemical analysis confirmed the expression of TL1A and its receptor DR3 by sarcoid pulmonary T cells infiltrating surgical pulmonary biopsies obtained from two patients with active sarcoidosis (Fig. 3a and b). When the cell sources of TL1A in sarcoid tissue was investigated, we showed that the cytokine was preferentially expressed by macrophage multinucleated giant cells and T cells in the granuloma, even if endothelial and metaplastic epithelial cells bore the two molecules. Immunohistochemical analysis of biopsies from one patient with refractory sarcoidosis and pulmonary fibrosis showed that lung T cells were mainly nonreactive for TL1A and DR3, whereas some epitheliod cells were weakly positive (Fig. 3c and d).

### TL1A triggers MMP-9 release by sarcoid alveolar macrophages

In presence of IFN-γ, TL1A increases MMP-9 production in the human monocytic cell line THP-1 [22]. Since IFN-γ is one of the prevalent cytokines in the lung of patients with active sarcoidosis [5], by gelatin zymography assays we evaluated MMP-9 activity in the lung of our patients.

**Fig. 4:**
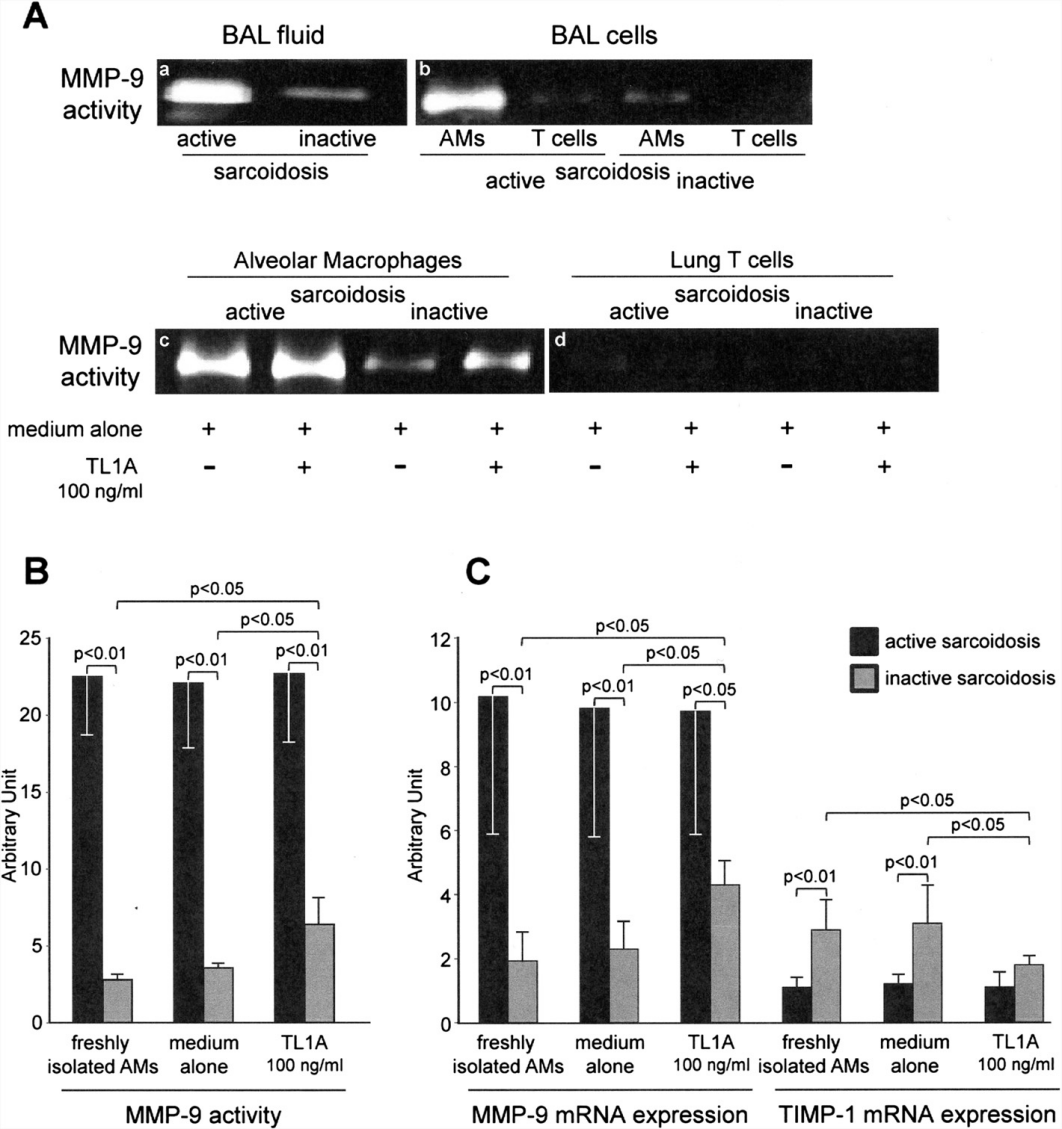
A. Gelatin zymograms of MMP-9 activity in the BAL fluid (**a**), and in freshly isolated BAL cellular components (alveolar macrophages and T cells) (**b**), of two representative cases of patients with active and inactive sarcoidosis. c, d) MMP-9 activity of alveolar macrophages (**c**) and lung T cells (**d**) obtained from the BAL of two representative cases of patients with active and inactive sarcoidosis, cultured in medium alone and with the cytokine TL1A. B) MMP-9 activity of alveolar macrophages obtained from the lung of patients affected by active sarcoidosis (black columns) and inactive sarcoidosis (*grey columns*), freshly isolated, cultured in medium alone and with TL1A. Zymographic band densities from all samples were quantified by densitometry. Data are expressed as mean ± SD. D) MMP-9 and TIMP-1 mRNA expression in alveolar macrophages obtained from the lung of patients affected by active sarcoidosis (black columns) and inactive sarcoidosis (grey columns), freshly isolated, cultured in medium alone and with TL1A. Expression of MMP-9 and TIMP-1 mRNAs was normalized on GAPDH mRNA. Data are expressed as mean ± SD

As shown in Fig. 4a, panel a, BAL fluids from patients with active sarcoidosis were characterized by a stronger MMP-9 activity (12.5 ± 5.8) as compared to those obtained from inactive sarcoidosis (3.6 % ± 1.3; *p* < 0.05). AM and T cell subset isolation (Fig. 4a, panel b) demonstrated that the source responsible of MMP-9 activity was represented by AMs (2.8 ± 0.35 and 22.5 ± 3.8 in AMs from inactive and active sarcoidosis, respectively; *p* < 0.01 vs active disease). Successively, purified AMs and T lymphocytes were cultured in presence (and absence) of TL1A to investigate its possible effects on MMP-9 production and activity. Figure 4a (panel c and d), and 4B showed that MMP-9 activity referable to lung T cells was not up-regulated by TL1A/DR3 interactions, whereas AMs obtained from patients with inactive sarcoidosis presented an appreciable increase of the metalloproteinase activity (3.56 ± 0.27 and 6.40 ± 1.75, in absence and presence of TL1A, respectively; *p* < 0.05). We did not detect any relevant change in MMP-9 activity of AMs derived from patients with active sarcoidosis in presence of TL1A (22.10 ± 4.03 and 22.73 ± 4.46, in absence and presence of TL1A, respectively; p: not significant), probably due to an already achieved peak of MMP-9 production and release by these strongly activated macrophages.

### Molecular analysis of MMP-9 and TIMP-1 expression

As shown in Fig. 4c, real-time PCR evaluation of MMP-9 gene expression by purified AMs demonstrated that MMP-9 mRNA expression levels were higher in freshly isolated AMs from patients with active sarcoidosis (10.17 ± 4.2) compared with the corresponding AMs from patients with the inactive form of the disease (1.93 ± 0.89; *p* < 0.01 vs active disease). As verified for MMP-9 activity, the presence of TL1A in the culture medium of AMs from patients with active sarcoidosis did not induce any variation in MMP-9 mRNA levels (9.8 ± 4.0 and 9.7 ± 3.9, in absence and presence of TL1A, respectively; p: not significant), whereas AMs recovered from patients with inactive sarcoidosis responded to TL1A stimulus, raising MMP-9 mRNA levels from 2.3 ± 0.8 to 4.3 ± 0.7 (in absence and presence of TL1A, respectively; *p* < 0.05).

Using real-time PCR, we evaluated the tissue inhibitor of metalloproteinase (TIMP)-1 gene expression by purified AMs of patients with active and inactive sarcoidosis (Fig. 4c). Interestingly, we found that AMs from patients with inactive sarcoidosis showed higher TIMP-1 mRNA amounts with respect to AMs from active disease (1.1 ± 0.3 and 2.9 ± 0.9, in inactive and active sarcoidosis, respectively; *p* < 0.01). When AMs were cultured with TL1A, TIMP-1 mRNA levels significant decreased only in AMs obtained from patients with inactive sarcoidosis (3.1 ± 1.1 and 1.8 ± 0.3, in absence and presence of TL1A, respectively; *p* < 0.05), whereas we did not point out any variation of TIMP-1 mRNA levels in AMs obtained from patients with active sarcoidosis (1.2 ± 0.3 and 1.1 ± 0.4, in absence and presence of TL1A, respectively; p: not significant).

## Discussion

In this report we provided evidence that cytokine TL1A and its receptor DR3 are expressed on pulmonary T cells and alveolar macrophages of patients with sarcoidosis, showing their highest expression in lung cells obtained from patients with the active form of the disease, and when localized around and inside the granuloma or, in general terms, at the sites of disease activity.

The pulmonary microenvironment of patients with sarcoidosis is characterized by a well-known highly polarized Th1/Th17 profile [1, 6], where cytokines such as IFN-γ, TNF-α, IL-2, IL-12, IL-17, IL-18, together with several chemokines, induce and maintain an inflammatory state further exacerbated by the constant recruitment of immune cells into the lung. Cell subsets and cytokine patterns dominating sarcoid lung environment are in line with immunologic conditions that are needed for the expression and the activity of the TL1A/DR3 system. In particular, TL1A expression is known to be stimulated by pro-inflammatory cytokines [21] which are known to be secreted in sarcoid lung; furthermore, DR3, usually mainly restricted to T lymphocytes [10], was expressed at high degree by T cells as sarcoid AMs.

TL1A engagement of DR3 results in functional signalling that may induce pro-inflammatory effects [21], such as memory CD4+ T cell secretion of pro-inflammatory cytokines (IFN-γ, TNF-α, IL-17) [23, 24], Th17 cell differentiation and proliferation [25], and the proliferation of regulatory T cells with an attenuated, although still debated, suppressive activity [26, 27]. These effects exactly reflect typical phenomena taking place in the lung during the sarcoid inflammatory process, which is characterized by a) the presence of activated CD45RO+/CD4+ T lymphocytes secreting pro-inflammatory cytokines and chemokines, b) the infiltration of Th17 cells functionally equipped and releasing IL-17 cytokine [1], and c) the increase of Treg cells that display poor suppressive capacity [28]. With this as a background we propose that the TL1A/DR3 axis may represent an important mediator of the chronic inflammation taking place in the lung of patients with sarcoidosis, similarly to what has been recently demonstrated in other Th1/Th17 chronic disorders as Chron’s disease, in which TL1A/DR3 account for IFN-γ and IL-17 increasing, intestinal Treg proliferation, and, in a murine model, for the development of collagen deposition [29].

We have also demonstrated that sarcoid AMs per se produce elevated levels of metalloproteinase (MMP)-9, a molecule belonging to a family of extracellular and cell surface-associated proteinases, implicated in tissue homeostasis, host defence and tissue repair also in sarcoidosis [30, 31]. Interestingly, TL1A and DR3 stimulation was able to increase the production of MMP-9 by AMs of patients with inactive sarcoidosis, while macrophages derived from the lung of patients with the active form of the disease did not vary MMP-9 production after TL1A stimulation. Since at basal conditions sarcoid AM per se show elevated MMP-9 production and activity, we hypothesize that AMs reached their plateau in terms of MMP-9 production and could not be further stimulated.

There are a number of data implicating MMPs in the development of tissue remodelling and fibrosis that may be observed in various inflammatory conditions [32, 33]. Fibrosis represents the irreversible phase of sarcoidosis [34]. In some patients, the persistence of the chronic inflammatory response, in addition to the failure of immune-regulatory mechanisms, leads to the invasion of pulmonary tissues by granulomas. In turn this favours the definitive derangement of alveolar structures with resultant fibrosis. In fact, sarcoid granulomas are known to promote a local exaggerated production of collagen and extracellular matrix, associated with an aberrant fibroblast migration and proliferation. The increasing of MMP-9 production induced by TL1A on sarcoid AMs might exacerbate these phenomenas contributing to the evolution of sarcoidosis towards its irreversible phase.

The MMP-9 high levels characterizing AMs from patients with active sarcoidosis paralleled the reduced mRNA amounts of its major inhibitor, TIMP-1. This suggests a shift in the delicate balance between the enzyme and its inhibitor, increasing this imbalance the risk of intra-alveolar fibrosis development. A similar condition marked by an increase in MMP-9 with respect to low levels of TIMP-1 has been linked to the asthma pathogenesis, particularly to explain the increased presence of submucosal fibrosis in asthmatic airways [35, 36]

## Conclusions

Our data highlight the presence of TL1A/DR3 axis in the lung of patients with sarcoidosis, particularly in those patients with the active form of the disease. On the basis of these findings it might be suggested that TL1A and DR3, favoring the production of MMP-9 by sarcoid AMs, further strengthen the inflammatory vicious loop take place in sarcoid lung, thus worsening the evolution of the disease and/or potentially delaying/compromising its resolution.

## Abbreviations

AMs: Alveolar macrophages
BAL: Bronchoalveolar lavage
CCL: Chemokine C-C motif ligand
CXCL: Chemokine C-X-C motif ligand
DLCO: Diffusing Capacity of the Lung for Carbon Monoxide
DR3: Death domain receptor 3
FCS: Fetal calf serum
FEV1: Forced Expiratory Volume in the first second
FVC: Forced vital capacity
HRP: Horseradish peroxidase
IFN: Interferon
IL: Interleukin
MMP-9: Matrix metalloproteinase 9
PBMC: Pheripheral blood mononuclear cell
Th: T helper
TIFF: Tiffeneau index
TIMP-1: Tissue inhibitor of metalloproteinase 1
TL1A: TNF-like ligand 1A

## References

[1] Facco M, Cabrelle A, Teramo A, Olivieri V, Gnoato M, Teolato S (2011). Sarcoidosis is a Th1/Th17 multisystem disorder. Thorax.

[2] Polverosi R, Russo R, Coran A, Battista A, Agostini C, Pomerri F (2014). Typical and atypical pattern of pulmonary sarcoidosis at high-resolution CT: relation to clinical evolution and therapeutic procedures. Radiol Med.

[3] Müller-Quernheim J, Prasse A, Zissel G (2012). Pathogenesis of sarcoidosis. Presse Med.

[4] Judson MA, Baughman RP (2014). Worsening of pulmonary sarcoidosis. Curr Opin Pulm Med.

[5] Agostini C, Trentin L, Facco M, Sancetta R, Cerutti A, Tassinari C (1996). Role of IL-15, IL-2, and their receptors in the development of T cell alveolitis in pulmonary sarcoidosis. J Immunol.

[6] Agostini C, Cabrelle A, Calabrese F, Bortoli M, Scquizzato E, Carraro S (2005). Role for CXCR6 and its ligand CXCL16 in the pathogenesis of T-cell alveolitis in sarcoidosis. Am J Respir Crit Care Med.

[7] Agostini C, Cassatella M, Zambello R, Trentin L, Gasperini S, Perin A (1998). Involvement of the IP-10 chemokine in sarcoid granulomatous reactions. J Immunol.

[8] Facco M, Baesso I, Miorin M, Bortoli M, Cabrelle A, Boscaro E (2007). Expression and role of CCR6/CCL20 chemokine axis in pulmonary sarcoidosis. J Leukoc Biol.

[9] Agostini C, Trentin L, Perin A, Facco M, Siviero M, Piazza F (1999). Regulation of alveolar macrophage-T cell interactions during Th1-type sarcoid inflammatory process. Am J Physiol.

[10] Aiba Y, Nakamura M (2013). The role of TL1A and DR3 in autoimmune and inflammatory diseases. Mediators Inflamm.

[11] Meylan F, Richard AC, Siegel RM (2011). TL1A and DR3, a TNF family ligand-receptor pair that promotes lymphocyte costimulation, mucosal hyperplasia, and autoimmune inflammation. Immunol Rev.

[12] Wallace KL, Zheng LB, Kanazawa Y, Shih DQ (2014). Immunopathology of inflammatory bowel disease. World J Gastroenterol.

[13] Cassatella MA, Pereira-da-Silva G, Tinazzi I, Facchetti F, Scapini P, Calzetti F (2007). Soluble TNF-like cytokine (TL1A) production by immune complexes stimulated monocytes in rheumatoid arthritis. J Immunol.

[14] Sun X, Zhao J, Liu R, Jia R, Sun L, Li X (2013). Elevated serum and synovial fluid TNF-like ligand 1A (TL1A) is associated with autoantibody production in patients with rheumatoid arthritis. Scand J Rheumatol.

[15] Li L, Fu L, Lu Y, Wang W, Liu H, Li F (2014). TNF-like ligand 1A is associated with the pathogenesis of psoriasis vulgaris and contributes to IL-17 production in PBMCs. Arch Dermatol Res.

[16] Bamias G, Jia LG, Cominelli F (2013). The tumor necrosis factor-like cytokine 1A/death receptor 3 cytokine system in intestinal inflammation. Curr Opin Gastroenterol.

[17] Kamada N, Hisamatsu T, Honda H, Kobayashi T, Chinen H, Takayama T (2010). TL1A produced by lamina propria macrophages induces Th1 and Th17 immune responses in cooperation with IL-23 in patients with Crohn's disease. Inflamm Bowel Dis.

[18] Roeleveld DM, Koenders MI. The role of the Th17 cytokines IL-17 and IL-22 in Rheumatoid Arthritis pathogenesis and developments in cytokine immunotherapy. Cytokine. 2014. doi:10.1016/j.cyto.2014.10.006. 10.1016/j.cyto.2014.10.006 25466295

[19] Bedoya SK, Lam B, Lau K, Larkin J (2013). Th17 cells in immunity and autoimmunity. Clin Dev Immunol.

[20] Agostini C, Trentin L, Zambello R, Bulian P, Siviero F, Masciarelli M (1993). CD8 alveolitis in sarcoidosis: incidence, phenotypic characteristics, and clinical features. Am J Med.

[21] Migone TS, Zhang J, Luo X, Zhuang L, Chen C, Hu B (2002). TL1A is a TNF-like ligand for DR3 and TR6/DcR3 and functions as a T cell costimulator. Immunity.

[22] Kang YJ, Kim WJ, Bae HU, Kim DI, Park YB, Park JE (2005). Involvement of TL1A and DR3 in induction of pro-inflammatory cytokines and matrix metalloproteinase-9 in atherogenesis. Cytokine.

[23] Bamias G, Mishina M, Nyce M, Ross WG, Kollias G, Rivera-Nieves J (2006). Role of TL1A and its receptor DR3 in two models of chronic murine ileitis. Proc Natl Acad Sci U S A.

[24] Kamada N, Hisamatsu T, Honda H, Kobayashi T, Chinen H, Takayama T (2010). TL1A produced by lamina propria macrophages induces Th1 and Th17 immune responses in cooperation with IL-23 in patients with Crohn's disease. Inflamm Bowel Dis.

[25] Jones GW, Stumhofer JS, Foster T, Twohig JP, Hertzog P, Topley N (2011). Naive and activated T cells display differential responsiveness to TL1A that affects Th17 generation, maintenance, and proliferation. FASEB J.

[26] Schreiber TH, Podack ER (2013). Immunobiology of TNFSF15 and TNFRSF25. Immunol Res.

[27] Taraban VY, Slebioda TJ, Willoughby JE, Buchan SL, James S, Sheth B (2011). Sustained TL1A expression modulates effector and regulatory T-cell responses and drives intestinal goblet cell hyperplasia. Mucosal Immunol.

[28] Oswald-Richter KA, Richmond BW, Braun NA, Isom J, Abraham S, Taylor TR (2013). Reversal of global CD4+ subset dysfunction is associated with spontaneous clinical resolution of pulmonary sarcoidosis. J Immunol.

[29] Bamias G, Jia LG, Cominelli F (2013). The tumor necrosis factor-like cytokine 1A/death receptor 3 cytokine system in intestinal inflammation. Curr Opin Gastroenterol.

[30] Agostini C, Garbisa S, Trentin L, Zambello R, Fastelli G, Onisto M (1989). Pulmonary alveolar macrophages from patients with active sarcoidosis express type IV collagenolytic proteinase. An enzymatic mechanism for influx of mononuclear phagocytes at sites of disease activity. J Clin Invest.

[31] Nissinen L, Kähäri VM (1840). Matrix metalloproteinases in inflammation. Biochim Biophys Acta.

[32] Vandenbroucke RE, Libert C (2014). Is there new hope for therapeutic matrix metalloproteinase inhibition?. Nat Rev Drug Discov.

[33] Henry MT, McMahon K, Mackarel AJ, Prikk K, Sorsa T, Maisi P (2002). Matrix metalloproteinases and tissue inhibitor of metalloproteinase-1 in sarcoidosis and IPF. Eur Respir J.

[34] Judson MA. The Clinical Features of Sarcoidosis: A Comprehensive Review. Clin Rev Allergy Immunol. 2014. [Epub ahead of print]. 10.1007/s12016-014-8450-y 25274450

[35] Mautino G, Capony F, Bousquet J, Vignola AM (1999). Balance in asthma between matrix metalloproteinases and their inhibitors. J Allergy Clin Immunol.

[36] Lee KS, Jin SM, Lee H, Lee YC (2004). Imbalance between matrix metalloproteinase-9 and tissue inhibitor of metalloproteinase-1 in toluene diisocyanate-induced asthma. Clin Exp Allergy.

